# Prospective study according to the SRS and SOSORT criteria on the effectiveness of a complete conservative treatment (bracing and exercises) for adolescent idiopathic scoliosis: efficacy and intent-to-treat analysis

**DOI:** 10.1186/1748-7161-8-S1-O36

**Published:** 2013-06-03

**Authors:** S Negrini, S Donzelli, F Zaina

**Affiliations:** 1 University of Brescia, Brescia, Italy; 2 IRCCS Don Gnocchi, Milan, Italy; 3 ISICO (Italian Scientific Spine Institute), Milan, Italy

## Background

A Cochrane review suggests that, beyond RCTs, studies according to the SRS and SOSORT criteria are tools to obtain evidence on the effectiveness of bracing for adolescent idiopathic scoliosis (AIS). Even though the SRS criteria propose to follow a prospective design, until now only one out of 6 published studies was prospective.

## Aim

Present prospective results of bracing completely following the SRS and SOSORT criteria.

## Methods

Study Design: Prospective - data extraction from a clinical database started in 2003. Population: According to SRS inclusion criteria (AIS, age 10 years or older; Risser test 0-2; Cobb degrees 25-40°; no prior treatment; less than one year post-menarche). 73 patients (60 females - 82.2% - and 13 males - 17.8%) have been included, age 12 years 10 months ± 17 months, with 39 single, 32 double, and 2 triple curves. Methods: Braces have been personalized (Sibilla 61.6%, Lyon 16.4%, Sforzesco 13.7%, SpineCor 6.8%). At the start of treatment, 30 patients used the brace 22-23 hours per day (h/d), 22 for 20-21 h/d, and 21 for 16-18 h/d; weaning was gradual after Risser 3; all patients performed exercises; SOSORT management criteria were respected. Outcome: SRS (unchanged; worsened 6° or more; over 45° at the end of treatment; surgically treated) and rate of improvement (6° or more); radiographic and clinical data. Analyses: overall results at the last evaluation of all patients. Intent-to-treat: failures included all drop-outs (treatment stopped before Risser 3, or without medical indication). Efficacy: end-of-treatment patients.

## Results

Median reported compliance for patients that completed the 3 years 4 months ± 20 months of treatment was 99.1% (range 22.2-109.2%). Overall, 7 (9.6%) patients worsened, 1 (1.4%) progressed beyond 45° and was fused; 46 patients (49.3%) improved, others were stable. Intent-to-treat: failures were 11 (15.1%); at start, they had statistically significant low BMI and kyphosis, high thoracic ATR and °Cobb, and they showed reduced compliance and years of treatment. Successes had statistically significant improvements in all parameters. Failures also improved, but not statistically.

## Conclusions

This study confirms the efficacy of conservative treatment respecting SOSORT criteria. Considering drop-outs as failures, even if they discontinued therapy with 22.7° (range 16-34°) scoliosis at Risser 1.3±1 stage, the rate of failure increased from 1.4% to 15.1%.
